# Bibliometric Analysis of the Top 100 Most Cited Papers in Mohs Surgery

**DOI:** 10.7759/cureus.62488

**Published:** 2024-06-16

**Authors:** Timothy E Nehila, Mohammad Tahseen Alkaelani, Bilal Koussayer, Fadia Fakhre, Taylor Blount, Meredith G Moore, Lilia Correa-Selm, Basil Cherpelis

**Affiliations:** 1 Department of Surgery, University of South Florida (USF) Health Morsani College of Medicine, Tampa, USA; 2 Department of Clinical Sciences, Florida State University College of Medicine, Tallahassee, USA; 3 Department of Surgery, University of Central Florida College of Medicine, Orlando, USA; 4 Department of Plastic Surgery, Florida State University College of Medicine, Tallahassee, USA; 5 Department of Plastic Surgery, University of South Florida (USF) Health Morsani College of Medicine, Tampa, USA; 6 Department of Dermatology, University of South Florida (USF) Health Morsani College of Medicine, Tampa, USA

**Keywords:** carcinoma, melanoma, oncology, mohs surgery, mohs

## Abstract

Background: Mohs micrographic surgery is a specialized tissue-sparing technique used to treat skin cancers.

Objective: By understanding the attributes that make a research paper one of the top 100 cited papers on Mohs surgery, we hope to illuminate seminal research in this field.

Methods and materials: The global literature about Mohs surgery published between 1900 and 2023 was searched on the Web of Science. Publication data for all results meeting the search criteria were exported and analyzed.

Results: In total, 4,961 publications with 81,405 citations were identified. Dermatologic Surgery was the most cited journal, with 1,073 publications. Papers from the top 100 most cited that were published in the year 2000 or prior had an average of 22.1 citations in the first five years after publication, whereas papers published after 2001 had an average of 56.0 citations in the first five years.

Conclusion: Analysis of the most cited papers on Mohs surgery demonstrates the influential role of the Dermatological Surgery journal in advancing the field. Noteworthy studies addressing cost, safety, and efficacy have received substantial citations, reflecting their significance within the literature. A trend toward more citation in the first five years after publication over time was identified.

## Introduction

Mohs micrographic surgery, named after pioneering surgeon Dr. Frederick Mohs, is a specialized tissue-sparing technique for the treatment of skin cancers. This method maximizes tissue preservation while offering complete microscopic margin control of the entire surgical margin. Over the years since the inception of Mohs surgery, numerous clinical and scientific papers on this excision method have been published in prestigious journals with varying impact factors. As the pace of scientific discovery has intensified, so has the pace and extent of publication, making it challenging to identify the most influential papers on the topic of Mohs surgery.

Citations play a crucial role in acknowledging the contributions of other authors and quantifying valuable work in a field. The number of citations received by an author's published work is often seen as an indicator of the article’s reputation and impact. This is similarly the case for scientific journals. The impact factor (IF) of a journal is a metric that reflects the average number of citations its articles receive [1]. It is calculated annually and serves as a proxy for the relative importance of an academic journal within its field. The Hirsch index (h-index) is defined as the number of papers with citation number ≥h and is another widely accepted quantitative method used to characterize the scientific output of a researcher or journal. A scientist or journal has an index h if h number of papers (Np) have at least h citations each and the other (Np - h) papers have ≤h citations each [2]. Journals with higher impact factors and higher h-indices are generally considered more influential.

Bibliometric citation analysis examines the citation history of published papers [3,4]. Various medical specialties have previously conducted analyses to identify the most cited papers in their respective fields [5,6]. These studies are beneficial in that they catalog works of significance which may be most beneficial for trainees, practitioners, and researchers alike. However, there has been no bibliometric analysis of the most cited papers on Mohs micrographic surgery to date.

In this study, we performed a bibliometric analysis on the most influential papers on Mohs surgery, based on their number of citations. By understanding the attributes that make a research paper one of the top 100 cited papers in the field of Mohs surgery, we hope to illuminate seminal research in this field. This analysis contributes to the existing body of knowledge and provides a reasonably comprehensive overview of the impactful contributions to Mohs surgery.

## Materials and methods

The global literature about Mohs surgery published between 1900 and 2023 was searched in English in the Web of Science (WOS) Core Collection platform (Clarivate PLC, London, England). The WOS platform is the most accepted citation database within the scientometric community [3, 7, 8, 9]. This query was performed on a single date (April 12, 2023) by a single researcher using predetermined keywords. The search terms used were "Mohs surgery" OR "Moh’s surgery" OR "Mohs’ surgery" OR "Mohs micrographic surgery" OR "Moh’s micrographic surgery" OR "Mohs’ micrographic surgery" and were intended to capture commonly used semantic variations. The data for all publications meeting this search criteria were exported in CSV format. The data downloaded from WOS included title, author(s), journal name, publication date, author affiliation, and total citations.

## Results

Our search returned 4,961 publications with a total of 81,405 citations. The average number of citations per paper for all publications meeting the search criteria was 16.4. Dermatologic Surgery was the most cited journal, with 1,073 publications. The Journal of the American Academy of Dermatology was second, with 559 publications (Table 1). The five-year impact factors for these journals as of May 2023 were 1.244 and 3.556, respectively [10,11].

**Table 1:** Top 10 journals publishing on Mohs surgery

Rank	Journal	Publications
1	Dermatologic Surgery	1073
2	Journal of the American Academy of Dermatology	559
3	British Journal of Dermatology	307
4	Journal of Dermatologic Surgery and Oncology	119
5	Australasian Journal of Dermatology	88
6	International Journal of Dermatology	86
7	Journal of Drugs in Dermatology	76
8	Archives of Dermatology (current JAMA Dermatology)	71
9	Dermatologic Clinics	64
10	Journal of the European Academy of Dermatology and Venereology	64

The top 100 most cited papers on Mohs surgery are listed in Table 2, arranged in a descending order based on the total number of citations. The total number of citations per article ranged between 115 and 981. The mean number of citations per paper in the top 100 was 198.2. The most recent paper in the top 100 was written in 2019 and has 249 citations [12]. The oldest paper in the top 100 was written in 1983 and has 178 citations [13]. The 2000s contributed the most papers of any decade to the top 100 most cited papers.

**Table 2:** Top 100 most cited papers on Mohs surgery

Rank	Title	Author	Journal	Year of publication	Total citations	Citations per year
1	Prognostic factors for local recurrence, metastasis, and survival rates in squamous-cell carcinoma of the skin, ear, and lip-implications for treatment modality selection	Rowe et al.	Journal of the American Academy of Dermatology	1992	981	30.66
2	Primary care: cutaneous squamous-cell carcinoma	Alam et al.	New England Journal of Medicine	2001	845	36.74
3	Current concepts-basal-cell carcinoma	Rubin et al.	New England Journal of Medicine	2005	543	28.58
4	Skin cancer in organ transplant recipients: epidemiology, pathogenesis, and management	Berg et al.	Journal of the American Academy of Dermatology	2002	493	22.41
5	Guidelines for the management of basal cell carcinoma	Telfer et al.	British Journal of Dermatology	2008	473	29.56
6	Mohs surgery is the treatment of choice for recurrent (previously treated) basal-cell carcinoma	Rowe et al.	Journal of Dermatologic Surgery and Oncology	1989	310	8.86
7	Surgical excision versus Mohs' micrographic surgery for primary and recurrent basal-cell carcinoma of the face: a prospective randomized controlled trial with 5-years' follow-up	Mosterd et al.	Lancet Oncology	2008	304	19
8	Dermatofibrosarcoma protuberans-a clinicopathologic analysis of patients treated and followed at a single institution	Bowne et al.	Cancer	2000	288	12
9	Malignant melanoma: prevention, early detection, and treatment in the 21st century	Rigel et al.	CA-A Cancer Journal for Clinicians	2000	283	11.79
10	AAD/ACMS/ASDSA/ASMS 2012 appropriate use criteria for Mohs micrographic surgery: a report of the American Academy of Dermatology, American College of Mohs Surgery, American Society for Dermatologic Surgery Association, and the American Society for Mohs Surgery	Connolly et al.	Journal of the American Academy of Dermatology	2012	276	23
11	Risk factors for cutaneous squamous cell carcinoma recurrence, metastasis, and disease-specific death a systematic review and meta-analysis	Thompson et al.	JAMA Dermatology	2016	271	33.88
12	Merkel cell carcinoma	Becker et al.	Nature Reviews Disease Primers	2017	261	37.29
13	Terahertz pulsed imaging of basal cell carcinoma ex vivo and in vivo	Wallace et al.	British Journal of Dermatology	2004	257	12.85
14	Metastatic basal-cell carcinoma-report of 12 cases with a review of the literature	Lo et al.	Journal of the American Academy of Dermatology	1991	256	7.76
15	Imatinib mesylate in advanced dermatofibrosarcoma protuberans: pooled analysis of two-phase II clinical trials	Rutkowski et al.	Journal of Clinical Oncology	2010	253	18.07
16	Guidelines of care for the management of primary cutaneous melanoma	Swetter et al.	Journal of the American Academy of Dermatology	2019	249	49.8
17	Guidelines of care for the management of primary cutaneous melanoma	Bichakjian et al.	Journal of the American Academy of Dermatology	2011	246	18.92
18	Surgical excision vs Mohs' micrographic surgery for basal-cell carcinoma of the face: randomized controlled trial	Smeets et al.	Lancet	2004	244	12.2
19	A systematic review of treatment modalities for primary basal cell carcinomas	Thissen et al.	Archives of Dermatology	1999	243	9.72
20	Dermatofibrosarcoma protuberans	Gloster	Journal of the American Academy of Dermatology	1996	242	8.64
21	Prognostic factors for metastasis in squamous cell carcinoma of the skin	Cherpelis et al.	Dermatologic Surgery	2002	234	10.64
22	A comparison between Mohs micrographic surgery and wide surgical excision for the treatment of dermatofibrosarcoma protuberans	Gloster et al.	Journal of the American Academy of Dermatology	1996	225	8.04
23	Interventions for basal cell carcinoma of the skin	Bath-Hextall et al.	Cochrane Database of Systematic Reviews	2007	221	13
24	Mohs micrographic surgery	Shriner et al.	Journal of the American Academy of Dermatology	1998	217	8.35
25	Sebaceous carcinoma	Nelson et al.	Journal of the American Academy of Dermatology	1995	214	7.38
26	Merkel cell carcinoma	Goessling et al.	Journal of Clinical Oncology	2002	207	9.41
27	Aggressive basal cell carcinoma: presentation, pathogenesis, and management	Walling et al.	Cancer and Metastasis Reviews	2004	206	10.3
28	Sebaceous carcinoma of the ocular region: a review	Shields et al.	Survey of Ophthalmology	2005	205	10.79
29	Diagnosis and treatment of basal cell carcinoma: European consensus-based interdisciplinary guidelines	Peris et al.	European Journal of Cancer	2019	203	40.6
30	Basal cell carcinoma of the face: surgery or radiotherapy? Results of a randomized study	Avril et al.	British Journal of Cancer	1997	202	7.48
31	The impact of in vivo reflectance confocal microscopy on the diagnostic accuracy of lentigo maligna and equivocal pigmented and nonpigmented macules of the face	Guitera et al.	Journal of Investigative Dermatology	2010	199	14.21
32	Mammary and extramammary Paget's disease	Kanitakis	Journal of the European Academy of Dermatology and Venereology	2007	196	11.53
33	Epidemiologic characteristics and clinical course of patients with malignant eyelid tumors in an incidence cohort in Olmsted County, Minnesota	Cook et al.	Ophthalmology	1999	193	7.72
34	A retrospective review of 1349 cases of sebaceous carcinoma	Dasgupta et al.	Cancer	2009	191	12.73
35	Mohs micrographic surgery for the treatment of dermatofibrosarcoma protuberans-results of a multiinstitutional series with an analysis of the extent of microscopic spread	Ratner et al.	Journal of the American Academy of Dermatology	1997	190	7.04
36	Adjuvant local irradiation for Merkel cell carcinoma	Lewis et al.	Archives of Dermatology	2006	187	10.39
37	The Roswell Park Cancer Institute experience with extramammary Paget's disease	Zollo et al.	British Journal of Dermatology	2000	185	7.71
38	Guidelines of care for the management of cutaneous squamous cell carcinoma	Alam et al.	Journal of the American Academy of Dermatology	2018	183	30.5
39	Multiprofessional guidelines for the management of the patient with primary cutaneous squamous cell carcinoma	Motley et al.	British Journal of Dermatology	2002	183	8.32
40	Treatment options and future prospects for the management of eyelid malignancies-an evidence-based update	Cook et al.	Ophthalmology	2001	182	7.91
41	Guidelines for the management of basal cell carcinoma	Telfer et al.	British Journal of Dermatology	1999	182	7.28
42	Management of nonmelanoma skin cancer in 2007	Neville et al.	Nature Clinical Practice Oncology	2007	180	10.59
43	Surgical excision versus Mohs' micrographic surgery for basal cell carcinoma of the face: A randomized clinical trial with 10 year follow-up	Van Loo et al.	European Journal of Cancer	2014	179	17.9
44	Guidelines of care for the management of basal cell carcinoma	Bichakjian et al.	Journal of the American Academy of Dermatology	2018	178	29.67
45	Lentigo maligna and lentigo meligna melanoma	Cohen	Journal of the American Academy of Dermatology	1995	178	6.14
46	Mohs surgery-technique, indications, applications, and the future	Swanson	Archives of Dermatology	1983	178	4.34
47	Mohs' micrographic surgery for treatment of basal cell carcinoma of the face-results of a retrospective study and review of the literature	Smeets et al.	British Journal of Dermatology	2004	176	8.8
48	Penile carcinoma: a challenge for the developing world	Misra et al.	Lancet Oncology	2004	172	8.6
49	Diagnosis of tumors during tissue-conserving surgery with integrated autofluorescence and Raman scattering microscopy	Kong et al.	Proceedings of the National Academy of Sciences of the United States of America	2013	171	15.55
50	Mohs micrographic surgery: a cost analysis	Cook et al.	Journal of the American Academy of Dermatology	1998	165	6.35
51	Extramammary Paget's disease: surgical treatment with Mohs micrographic surgery	Hendi et al.	Journal of the American Academy of Dermatology	2004	164	8.2
52	Mohs micrographic surgery for the treatment of primary cutaneous melanoma	Zitelli et al.	Journal of the American Academy of Dermatology	1997	163	6.04
53	Dermatofibrosarcoma protuberans: a report on 29 patients treated by Mohs micrographic surgery with long-term follow-up and review of the literature	Snow et al.	Cancer	2004	162	8.1
54	Vulvar lichen sclerosus and squamous cell carcinoma: a cohort, case control, and investigational study with historical perspective; Implications for chronic inflammation and sclerosis in the development of neoplasia	Carlson et al.	Human Pathology	1998	161	6.19
55	Current treatment options in dermatofibrosarcoma protuberans	Lemm et al.	Journal of Cancer Research and Clinical Oncology	2009	160	10.67
56	Reflectance confocal microscopy for in vivo skin imaging	Calzavara-Pinton et al.	Photochemistry and Photobiology	2008	159	9.94
57	Metastatic basal-cell carcinoma-report of 5 cases	Snow et al.	Cancer	1994	159	5.3
58	Cutaneous squamous cell carcinoma treated with Mohs micrographic surgery in Australia I. Experience over 10 years	Leibovitch et al.	Journal of the American Academy of Dermatology	2005	153	8.05
59	Local control of primary Merkel cell carcinoma: review of 45 cases treated with Mohs micrographic surgery with and without adjuvant radiation	Boyer et al.	Journal of the American Academy of Dermatology	2002	151	6.86
60	Preservation of RNA for functional genomic studies: a multidisciplinary tumor bank protocol	Florell et al.	Modern Pathology	2001	151	6.57
61	Update of the European guidelines for basal cell carcinoma management developed by the Guideline Subcommittee of the European Dermatology Forum	Trakatelli et al.	European Journal of Dermatology	2014	149	14.9
62	Update on keratinocyte carcinomas	Nehal et al.	New England Journal of Medicine	018	148	24.67
63	Dermatofibrosarcoma protuberans: a comprehensive review and update on diagnosis and management	Llombart et al.	Seminars in Diagnostic Pathology	2013	146	13.27
64	Diagnosis of nonmelanoma skin cancer/keratinocyte carcinoma: a review of diagnostic accuracy of nonmelanoma skin cancer diagnostic tests and technologies	Mogensen et al.	Dermatologic Surgery	2007	145	8.53
65	Outcomes of surgery for dermatofibrosarcoma protuberans	Chang et al.	EJSO	2004	143	7.15
66	Skin cancer in solid organ transplant recipients: advances in therapy and management Part II. Management of skin cancer in solid organ transplant recipients	Zwald et al.	Journal of the American Academy of Dermatology	2011	142	10.92
67	Dermatofibrosarcoma protuberans	Mendenhall et al.	Cancer	2004	142	7.1
68	Low recurrence rate after surgery for dermatofibrosarcoma protuberans-a multidisciplinary approach from a single institution	DuBay et al.	Cancer	2004	142	7.1
69	Confocal examination of nonmelanoma cancers in thick skin excisions to potentially guide Mohs micrographic surgery without frozen histopathology	Rajadhyaksha et al.	Journal of Investigative Dermatology	2001	139	6.04
70	Merkel cell carcinoma-comparison of Mohs micrographic surgery and wide excision in eighty-six patients	OConnor et al.	Dermatologic Surgery	1997	139	5.15
71	Interventions for non-metastatic squamous cell carcinoma of the skin: systematic review and pooled analysis of observational studies	Lansbury et al.	BMJ-British Medical Journal	2013	137	12.45
72	Microcystic adnexal carcinoma-forty-eight cases, their treatment, and their outcome	Chiller et al.	Archives of Dermatology	2000	136	5.67
73	Basal-cell carcinoma of the eyelid and periocular skin	Margo et al.	Survey of Ophthalmology	1993	136	4.39
74	Lentigo maligna/lentigo maligna melanoma: current state of diagnosis and treatment	McKenna et al.	Dermatologic Surgery	2006	133	7.39
75	Cutaneous head and neck melanoma treated with Mohs micrographic surgery	Bricca et al.	Journal of the American Academy of Dermatology	2005	131	6.89
76	Dermatofibrosarcoma protuberans: wide local excision vs. Mohs micrographic surgery	Paradisi et al.	Cancer Treatment Reviews	2008	130	8.13
77	Opioid pain medication use after dermatologic surgery a prospective observational study of 212 dermatologic surgery patients	Harris et al.	JAMA Dermatology	2013	129	11.73
78	Basal cell skin cancer, version 1.2016	Bichakjian et al.	Journal of the National Comprehensive Cancer Network	2016	127	15.88
79	A prospective evaluation of the incidence of complications associated with Mohs micrographic surgery	Cook et al.	Archives of Dermatology	2003	127	6.05
80	Margin control for lentigo-maligna	Robinson	Journal of the American Academy of Dermatology	1994	126	4.2
81	Surgical margins for melanoma in situ	Kunishige et al.	Journal of the American Academy of Dermatology	2012	125	10.42
82	Surgical margins for excision of dermatofibrosarcoma protuberans	Parker et al.	Journal of the American Academy of Dermatology	1995	124	4.28
83	Confocal mosaicing microscopy in Mohs skin excisions: feasibility of rapid surgical pathology	Gareau et al.	Journal of Biomedical Optics	2008	123	7.69
84	A review of laser and photodynamic therapy for the treatment of nonmelanoma skin cancer	Marmur et al.	Dermatologic Surgery	2004	122	6.1
85	Surgical monotherapy versus surgery plus adjuvant radiotherapy in high-risk cutaneous squamous cell carcinoma: a systematic review of outcomes	Jambusaria-Pahlajani et al.	Dermatologic Surgery	2009	121	8.07
86	Sentinel lymph node biopsy in cutaneous squamous cell carcinoma: a systematic review of the English literature	Ross et al.	Dermatologic Surgery	2006	121	6.72
87	Complications of cutaneous surgery in patients who are taking warfarin, aspirin, or nonsteroidal anti-inflammatory drugs	Otley et al.	Archives of Dermatology	1996	121	4.32
88	Extramammary Paget's disease: summary of current knowledge	Lam et al.	Dermatologic Clinics	2010	120	8.57
89	Basal cell carcinoma treated with Mohs surgery in Australia - II. Outcome at 5-year follow-up	Leibovitch et al.	Journal of the American Academy of Dermatology	2005	120	6.32
90	Excision margins for nonmelanotic skin cancer	Thomas et al.	Plastic and Reconstructive Surgery	2003	120	5.71
91	Mohs micrographic surgery for lentigo maligna and lentigo maligna melanoma-a follow-up study	Cohen et al.	Dermatologic Surgery	1998	120	4.62
92	The role of radiation therapy in the management of dermatofibrosarcoma protuberans	Ballo et al.	International Journal of Radiation Oncology Biology Physics	1998	119	4.58
93	Photodynamic therapy with topical methyl aminolaevulinate for 'difficult-to-treat' basal cell carcinoma	Vinciullo et al.	British Journal of Dermatology	2005	118	6.21
94	Management of lentigo maligna and lentigo maligna melanoma with staged excision-a 5-year follow-up	Bub et al.	Archives of Dermatology	2004	118	5.9
95	Perineural invasion of cutaneous malignancies	Feasel et al.	Dermatologic Surgery	2001	118	5.13
96	Intraoperative and postoperative bleeding problems in patients taking warfarin, aspirin, and nonsteroidal antiinflammatory agents-a prospective study	Billingsley et al.	Dermatologic Surgery	1997	118	4.37
97	What is the best surgical margin for a basal cell carcinoma: a meta-analysis of the literature	Gulleth et al.	Plastic and Reconstructive Surgery	2010	116	8.29
98	The Australian Mohs database, part II-periocular basal cell carcinoma outcome at 5-year follow-up	Malhotra et al.	Ophthalmology	2004	116	5.8
99	Tumor recurrence 5 years after treatment of cutaneous basal cell carcinoma and squamous cell carcinoma	Chren et al.	Journal of Investigative Dermatology	2013	115	10.45
100	Predictors of extensive subclinical spread in nonmelanoma skin cancer treated with Mohs micrographic surgery	Batra et al.	Archives of Dermatology	2002	115	5.23

Regarding author location, 14 countries were responsible for producing the top 100 most cited papers. The United States of America produced most of these papers (70%), followed by the United Kingdom (6%), Australia (5%), and The Netherlands (5%) (Table 3). The Mayo Clinic (6%) and The University of Michigan (6%) were the institutions which contributed most to the top 100 most cited papers on Mohs surgery (Table 4).

**Table 3:** The countries of origin of the top 100 papers on Mohs surgery

Country	Number of publications
United States of America	70
United Kingdom	6
Australia	5
Netherlands	5
Italy	3
Germany	2
France	2
Canada	1
Denmark	1
Greece	1
India	1
New Zealand	1
Poland	1
Spain	1

**Table 4:** The institutions which contributed the most publications to the top 100 papers on Mohs surgery

Rank	Institution	Number of publications
1	Mayo Clinic	6
2	University of Michigan	6
3	Maastricht University	5
4	University of Texas Health Science Center	4
5	University of Wisconsin-Madison	4
6	Adelaide University	3
7	Columbia University	3
8	Harvard University	3
9	Nottingham University	3
10	Memorial Sloan-Kettering Cancer Center	3

Articles with a topic specifically related to Mohs surgery by title rather than a general disease process (i.e., cutaneous malignancy) comprised 25 of 100, whereas the remaining 75% mention Mohs micrographic surgery as a treatment modality but are not focused on this technique.

A large proportion of the top 100 cited papers specifically set out to compare Mohs to an alternative or more traditional excision method; 24% of papers were comparison study case series, and another 7% of papers were comparison systematic reviews or metanalyses. A total of 25% of papers were review articles on either Mohs surgery or pathologies typically treated with Mohs surgery, and 11% of papers were published guidelines on the implementation of Mohs surgery.

The most cited paper meeting our search criteria was a systematic review and metanalysis of all studies from 1940 to 1992 on the prognosis of squamous cell carcinoma of the skin and lip. It found that local recurrences occur less frequently when treated with Mohs micrographic surgery. It was published in 1992 and has 981 citations [14], 38 of those 981 citations (3.87%) came in the first five years after publication. The second most cited paper was a review article for the New England Journal of Medicine on cutaneous squamous cell carcinoma. It was published in 2001 and has 845 citations [15]. A total of 63 of those 845 citations (7.46%) came in the first five years after publication.

**Figure 1:**
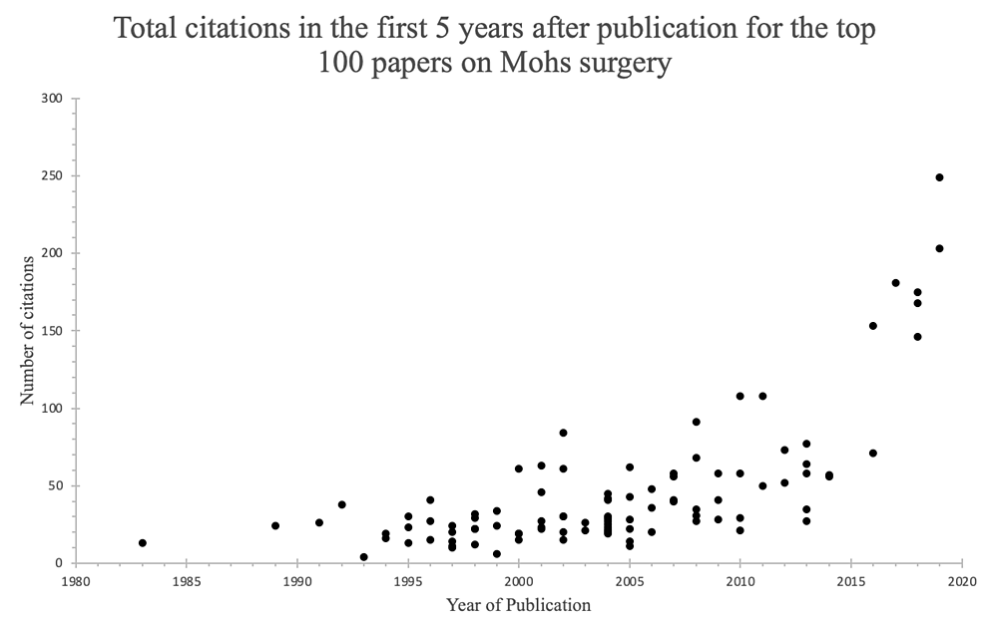
Total citations in the first five years after publication for the top 100 most cited papers on Mohs surgery

There was a trend towards more citation in the first 5 years after publication over time. Papers from the top 100 that were published in the year 2000 or prior had an average of 22.1 citations in the first five years after publication, whereas papers in the top 100 published after 2001 had an average of 56.0 citations in the first five years after publication (Figure 1).

Another landmark systematic review and meta-analysis on the risk factors associated with recurrence of cutaneous squamous cell carcinoma, published in 2016, was the 11th most cited paper with 271 citations. This paper found that tumor depth is associated with the highest rate of local recurrence and metastasis, and tumor diameter exceeding 20 mm is associated with the highest rate of disease specific death [16]. Although this paper did not specifically look at patients undergoing Mohs surgery, these findings are both applicable to the field and evidence for the efficacy of Mohs surgery.

Other landmark papers on Mohs surgery for the treatment of dermatofibrosarcoma protuberans, basal cell carcinoma, and extramammary Paget’s disease were ranked eighth, 44th and 51st, respectively [17-19].

## Discussion

The analysis of landmark papers among the top 100 most cited in Mohs surgery revealed several key findings. Notably, studies addressing the cost, safety, and efficacy of Mohs surgery were prominent. For instance, Cook et al.'s study in 1998, which investigated the cost of Mohs surgery, ranked as the 50th most cited article with 165 citations [20]. Similarly, Connolly et al.'s paper from 2012, focusing on appropriate use criteria for Mohs surgery, garnered 276 citations, emphasizing the importance of safety considerations [21]. Additionally, Rowe's meta-analysis in 1992, which assessed the efficacy of Mohs surgery for squamous cell carcinoma, stood out as the most cited paper with 981 citations [14]. These influential studies demonstrate that key research supporting the use and application of Mohs surgery continues to be highly cited years after publication.

While it is expected that highly cited papers would tend to be older, a phenomenon known as "obsolescence by incorporation" may explain the absence of Merritt et al.'s landmark 2012 multicenter study on the safety of Mohs surgery from the top 100 most cited papers [22]. This study, highlighting patient safety considerations in Mohs surgery, may have been widely accepted and swiftly incorporated into subsequent research, leading to fewer explicit citations.

One important finding from our analysis is the considerable impact of the Dermatological Surgery journal in the field of Mohs surgery. This is the closest to a field-specific scientific journal as there is no publication dedicated to micrographic surgery specifically. Many papers published in this journal garnered significant citation counts, signifying their influence and relevance within the literature. As of 2023, the Dermatological Surgery journal is the 31st highest-ranking journal in the field of dermatology by impact factor, and the fifth highest ranking by h-index [23,24]. However, our analysis reveals that it is by far the most impactful journal in the field of Mohs surgery. This discrepancy highlights that viewing a journal’s impact within an entire discipline may diminish the nuanced impact that each academic journal has on individual specialties within the disciplines they serve. Further, articles published in an esteemed journal may garner more citations due to their perceived importance, regardless of content.

Another interesting finding from our analysis was the trend towards more citations in the first five years after publication over time (Figure 1). This trend may be explained by topic growth. One previous study on topic growth and citation impact demonstrated that publications in fast-growing topics have a citation advantage compared to publications in slow-growing or declining topics and that using citations as a proxy for research evaluation may give incentives for researchers to publish in fast-growing topics [25]. Our analysis supports these findings. Another reason for this trend could be the general increase in scholarly activity over time, with the number of active journals increasing between 3.3% and 4.7% per year from 1900 to 1996 [26]. This general trend toward more scholarly volume over time is specifically corroborated by our findings in Mohs surgery as evidenced by Figure 1, which shows a trend toward more citation within the first five years after publication in papers written between 2015 and 2020 as compared to older publications.

It is important to acknowledge certain limitations of bibliometric analysis. First, our search methodology was necessarily broad and thus included papers that were only tangentially related to Mohs surgery or were smaller studies that probably would not be included in a more curated top 100 list. Papers of this sort included in our top 100 focused on topics like the broad management of cutaneous melanoma and Paget’s disease or were small scale case reports. These papers were ranked 16th, 32nd, 37th, and 57th in our top 100 [12,27-29].

Further, there are several biases associated with bibliometric analysis. First, self-citation may introduce bias, as authors may cite their own previously published work more frequently. Additionally, the citation count of a paper is influenced by time dependency, with older papers generally having more citations due to their longer period of availability for citation. Moreover, it is essential to recognize potential language and geographic biases in bibliometric analyses. The predominant use of English-language databases may overlook non-English publications and research from specific regions or countries. Therefore, generalizations about the field should be made with caution.

Finally, it is important to acknowledge the limitations of using citations as a sole measure of impact. While citations reflect academic influence and recognition, they may not capture other forms of impact, such as societal or industry influence, policy impact, or practical applications. A comprehensive assessment of research impact should consider multiple factors as a high citation count does not guarantee high-quality research.

## Conclusions

In conclusion, our analysis of the 100 most cited papers on Mohs surgery demonstrates the influential role of the Dermatological Surgery journal in advancing the field and catalogs highly referenced works in the field. Noteworthy studies addressing cost, safety, and efficacy have received substantial citations, reflecting their significance within the literature. However, it is crucial to consider the limitations of bibliometric analysis, including time dependency, self-citation, potential biases, and the need for a holistic assessment of impact beyond citations.
